# Defense guard: strategies of plants in the fight against Cadmium stress

**DOI:** 10.1007/s44307-024-00052-6

**Published:** 2024-12-02

**Authors:** Qian-hui Zhang, Yi-qi Chen, Zhen-bang Li, Xuan-tong Tan, Guo-rong Xin, Chun-tao He

**Affiliations:** 1 State Key Laboratory of Biocontrol, Guangdong Provincial Key Laboratory of Plant Stress Biology, School of Agriculture and Biotechnology, Shenzhen Campus of Sun Yat-Sen University, Sun Yat-Sen University, Shenzhen City, 518107 China; 2 Instrumental Analysis & Research Center, Guangdong Province, Sun Yat-Sen University, Guangzhou City, 510275 China

**Keywords:** Cadmium, Cd uptake, Cd transportation, Cd detoxification, Cd resistance, Cd-pollution safety cultivar

## Abstract

Soil Cadmium (Cd) contamination is a worldwide problem with negative impacts on human health. Cultivating the Cd-Pollution Safety Cultivar (Cd-PSC) with lower Cd accumulation in edible parts of plants is an environmentally friendly approach to ensure food security with wide application prospects. Specialized mechanisms have been addressed for Cd accumulation in crops. This review provides an extensive generality of molecular regulation mechanisms involved in Cd absorption, transport, detoxification, and tolerance in plants, highlighting key aspects of rhizosphere, apoplast barrier, Cd uptake, transfer, and cellular repair strategies under Cd stress. Additionally, we summarize the possible approaches for lowering the Cd accumulation crops, including molecular-assistant breeding, applying chemical materials, and microbial strategy to decrease Cd content in edible parts and improve Cd tolerance of crops under Cd stress. This review would provide valuable insights for cultivating low Cd accumulated crop cultivars, ultimately contributing to food safety.

## Introduction

Cadmium (Cd) is a non-essential and poisonous element of environmental concerns, which has induced environmental risks, especially in soil with agricultural and industrial activities (Wang et al 2021). All organisms, including plants, face with the challenge of accidental Cd biomagnification and bioaccumulation (Notariale et al 2021). The plant growth is inhibited when constantly exposed to Cd stress, resulting in reductions in plant yield and overall productivity (Rizwan et al 2016). Moreover, the Cd contaminations in crops threatens human health by consuming Cd-contaminated crops (Sharma et al 2021).

Plants have developed different ecotypes, including Cd hyper-accumulative ecotypes, and Cd resistance ecotypes, Cd sensitive ecotypes involving intricate metal homeostasis regulation strategies for adaptation to extreme metallic environments (Xiong et al 2004; Szopiński et al 2020). Previous studies have made essential progress in elucidating Cd transport and tolerance mechanisms in plants, including Cd deposition in the cell wall, Cd absorption, efflux and transportation, Cd chelation and vacuolar sequestration, and detoxification system (Zhang et al 2022b; Ghori et al 2019). Nevertheless, plants with different Cd accumulations emerge with diverse performances in specific processes. For instance, more Cd is detained in the roots cell walls of Huajun 2 (low-Cd *Brassica chinensis L.* cultivar) than that of Hanlv (high-Cd *Brassica chinensis L. cultivar*), which is resulted from lower degree of methyl-esterification (DM) pectin and higher pectin methylesterase (PME) activity in Huajun 2 than those in Hanlv (Wang et al 2020). In addition, higher Cd distributed in root phytochelatin fraction in C13 (High-Cd accumulated *Lactuca sativa*) than that in C16 (Low-Cd accumulated *Lactuca sativa*) may be due to the distinct glutathione supply for phytochelatins synthesis under Cd stress (He et al., 2024). These findings highlight the diverse approaches adopted in underlying Cd accumulation in edible parts of plants.

Therefore, it is vital to comprehend intricate mechanisms controlling Cd uptake, transport, accumulation, and detoxification in plants, which would be instrumental in enhancing Cd tolerance, reducing Cd accumulations, and minimizing the risk of consuming Cd-contaminated crops (Keyster et al 2020). This review aims to offer new insights into crop improvement strategies to enhance Cd tolerance and decrease Cd accumulation in the edible parts of crops.

## The molecular mechanisms of Cd tolerance and accumulation in plants

The mechanisms underlying Cd tolerance and accumulation in plants encompass several key processes, including root absorption, transportation, vacuole storage, and detoxification.

### Root exudates and rhizosphere microorganism

Recent studies have identified that various root exudates contribute to Cd tolerance and Cd detoxification in plants. For example, Cd stress induces the secretion of 20 root exudate compounds in the hyper accumulator *Sedum alfredii* (Luo et al 2014). Certain secondary metabolites contribute to Cd stabilization in soil (Bali et al., 2020). Additionally, soil microorganisms can also produce organic acids and plant hormones, in which anionic functional groups can decrease the Cd phyto-availability through chelation (El Rasafi et al 2022). Endophytic and rhizosphere microorganisms, including fungi and bacteria, are crucial for enhancing metal tolerance in plants (Raklami et al 2022; Halim et al 2020). For example, the Cd-resistant fungal strain *Penicillium janthinellum* (ZZ-2) exhibits a high capacity for Cd^2+^ absorption. Inoculation bermudagrass with ZZ-2 greatly improves Cd uptake by generating indole-3-acid (IAA), which mitigates Cd toxicity and promotes plant growth under Cd stress (Xie et al 2021). The endophytic fungus (*Neotyphodium coenophialum*) facilitates Cd accumulation and root-to-shoot transportation, which would promote the growth of tall fescue (*Lolium arundinaceum*) under Cd stress (Ren et al 2011). The colonization of arbuscular mycorrhizal fungi (AMF) promoted plant Cd tolerance by inducing metal detoxification related genes expression in plants. For instance, *rhizophagus irregularis* AMF could promote the expressions of *PtMT2b* in roots of *Populus trihocarpa* associated with metallothionein*.* AMF decreases the Cd accumulation in the leaves by 40%, presumably increasing intracellularly Cd binding and limiting Cd translocation to the shoot (Podar & Maathuis 2022).

### Apoplast barrier

**Fig. 1:**
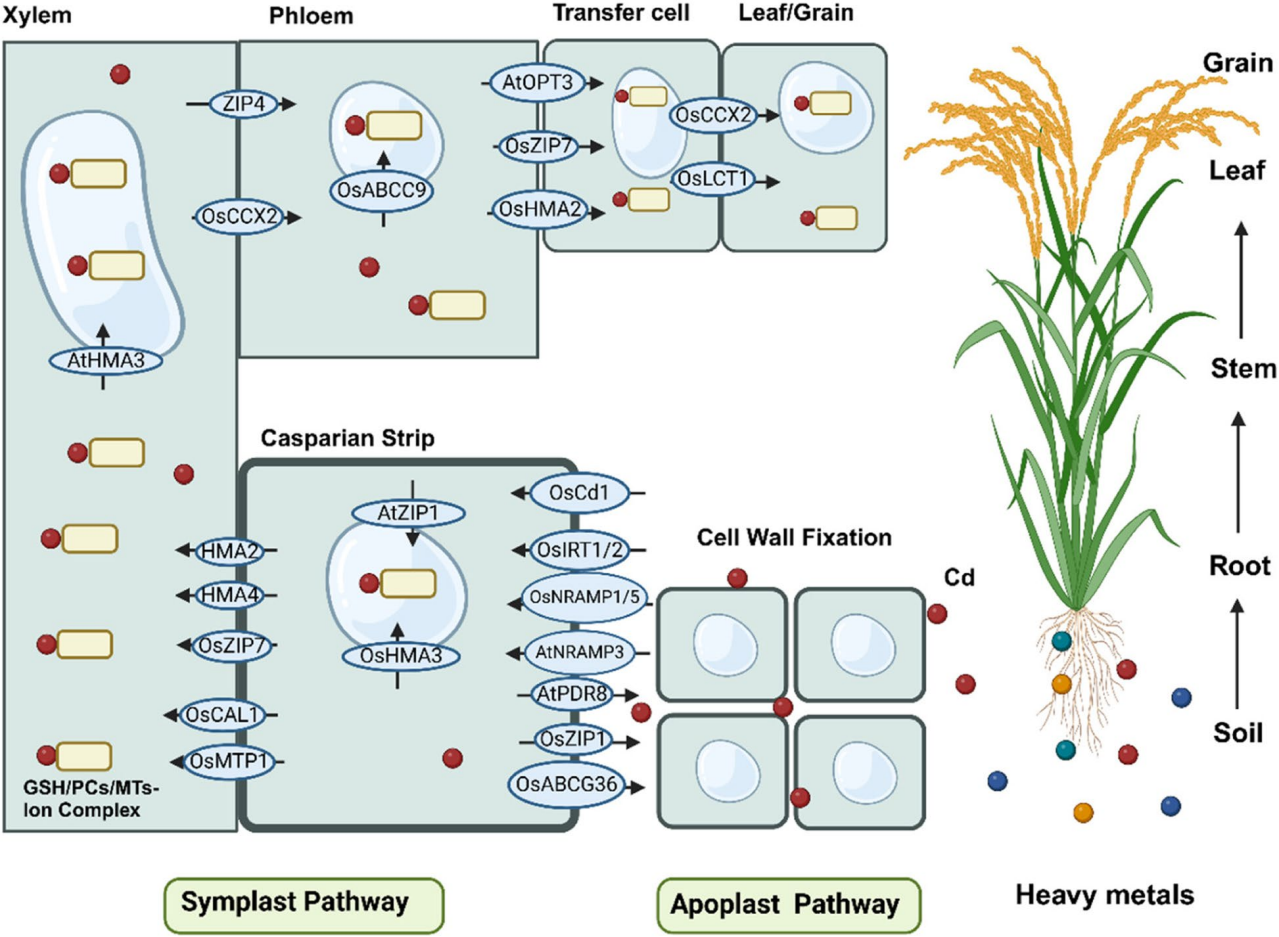
The process of Cd uptake, efflux, translocation, and distribution in plants. Cd is absorbed by the roots from the soil through both the apoplast and symplast pathways. Transporters are involved in the absorption and transport of Cd from soils to edible parts in plants. The pathways of Cd accumulation and transport include Cd fixation in the cell wall, Cd uptake and efflux by transporters, Cd chelation and vacuoles sequestration, root-to-shoot translocation, and redistribution between through stems and nodes, and further translocated to leaves and grains through the phloem

The cell wall serves as the first barrier in response to adverse Cd contamination (Wei et al., 2023; Parrotta et al., 2015). The root cell wall plays a crucial role in the contact of Cd and root surface cells. The cell wall helps prevent excessive Cd from entering the symplast by attaching a part of Cd2 + into cell wall, thereby improving Cd retention in roots and reducing Cd content in the shoot of rice (Nocito et al., 2011; Loix et al., 2017) (Fig. 1).

The plant cell wall is mainly composed of polysaccharides (pectin, cellulose, hemicellulose, lignin, callose) and proteins. All cell wall components contain functional groups like –OH, –COOH, and –SH, which can bind to the Cd^2+^, effectively immobilizing and inactivating Cd (Fig. 1). Therefore, the capacity of plants to Cd accumulation mostly depends on the Cd fixation in cell wall. For example, up to 70% of Cd is bound to the cell wall of the *Elodea canadensis* (De Caroli et al 2020). Similarly, about 73–83% of the Cd in *Salix matsudana* roots has accumulated in the cell walls, with a significant increase in the thickness of root xylem cell walls (Yu et al 2023). Approximately 76–80% of Cd is presented in the cell-wall fraction of the non-hyperaccumulating ecotype (NHE) of *Sedum alfredii* (Li et al 2015).

Cell wall thickening is a common response to prevent Cd from entering the protoplast under Cd stress. The root cell wall of *Vicia faba* thickens exposed to Cd and lead (Pb), which facilitates the heavy metal deposition and avoids direct cell damage (Nadgórska-Socha et al 2013; Alle 2019). The concentrations of pectin, hemicellulose 1, and hemicellulose 2 in roots of NHE *S. alfredii* increased by 35.6, 37.4, and 26.9% under Cd treatment compared with the control (Li et al 2015). The lignin in cell wall is effective for heavy metal ions sequestering, and enhanced lignin biosynthesis is ubiquitous in response to Cd exposure. For example, the xyloglucan, catalyzed by the xyloglucan endotransglucosylase/hydrolase (XTH), influences the Cd accumulation by influencing the Cd binding site in root cell wall. Overexpression of *PeXTH* in *Populus euphratica* facilitates the decomposition of xyloglucan and decrease its content in the cell wall, hence reducing the Cd content of the cell wall (Zhang et al 2022b).

### Cd uptake and transport in crops

Cd and essential divalent ions like ferrum (Fe), zinc (Zn), calcium (Ca), and other essential nutrient elements have similar physicochemical properties, which allows Cd to be absorbed into root cells and transported to other tissues via membrane transporters for essential and beneficial nutrient elements (Zhao et al 2022). The physicochemical similarities between Cd and these essential elements would constrain transporter proteins to distinguish between highly similar substrate ions (Sterckeman & Thomine 2020). As a result, the uptake and transportation of Cd, along with other heavy metals, are often carried out by the membrane transporters for essential elements (Yang et al 2022b). Cd transportation and accumulation have been extensively investigated in crops (Li et al., 2023a) (Fig. 1). Metal transporters involved in Cd uptake and transport include zinc (Zn)-/iron-regulated transporter-like proteins (ZIP), natural resistance-associated macrophage protein (NRAMP), iron (Fe)-regulated transporter (IRT), heavy metal ATPase (HMA), as well as yellow stripe-like protein (YSL) (Li et al., 2023a).

#### Cd uptake and efflux by transporters in roots

Cd uptake is achieved through plasma membrane transporters involved in the uptake of other nutrient elements, including Ca, magnesium (Mg), Fe, manganese (Mn), and Zn (Li et al., 2023a) (Fig. 1). Both Mn transporter NRAMP1 and the Fe (II) transporter IRT1 are capable of absorbing and transporting Cd in *Arabidopsis* (Zhao et al 2022). OsIRT1 and OsIRT2 are crucial for Cd uptake in rice (Zhang et al 2022b). After the application of sufficient Fe and Cd in cultivation solution, the expression of *OsIRT1* is significantly increased in rice roots (Hussain et al 2020). Yeast mutants expressing the rice Fe2 + transporter OsIRT1 and OsIRT2 become more sensitive to Cd, confirming that OsIRT1 and OsIRT2 are involved in Cd absorption in rice (Nakanishi et al 2006). The transgenic plant overexpressing *OsIRT1* is more sensitive to CdCl_2_ (Lee & An 2009). OsNRAMP5 is another membrane transporter responsible for the Cd transportation in rice (Sasaki et al 2012). The *osnramp5* knockout mutant significantly reduces Cd and Mn concentrations and increases Cd tolerance (Sasaki et al 2012). The tandem duplication of *OsNramp5* is responsible for low-Cd accumulation in a rice cultivar *Pokkali*, and introgression this allele into rice cultivar *Koshihikari* decreased Cd accumulation in grains significantly (Yu et al 2022). Similarly, the HvNramp5 transporter participates in Cd and Mn uptake rather than Fe in *Hordeum vulgare* (Wu et al 2016). OsNRAMP1 is highly homologous to OsNRAMP5, which is involved in Cd and Mn uptake and transport in rice (Chang et al 2020). The knockout of *OsNRAMP1* significantly decreases Cd and Mn uptake in roots and their accumulations in rice shoots and grains (Chang et al 2020).

The concentrations of Cd, Zn, and Mn are considerably increased in tobacco shoots overexpressing *SaNRAMP1*, indicating that SaNRAMP1 is a new NRAMP protein involved in Cd and Zn accumulation in *S. alfredii* (Zhang et al 2020a). A member of the major facilitator superfamily (MFS) *OsCd1* is expressed on the plasma membrane of rice roots (Zhang et al 2022b). *OsCd1* is involved in root Cd uptake and grain Cd accumulation in rice, while the *indica* variety carrying the *japonica* allele *OsCd1* ^*V449*^ can reduce grain Cd accumulation (Yan et al 2019). The *OsCd1* mutation significantly reduces the efficiency of Cd absorption in rice and Cd accumulation in grains (Chen et al 2023).

Different from the influxes of IRT and NRAMP families, OsZIP1, OsZIP3, AtPDR8, and OsABCG36 mainly influence Cd accumulation through the Cd efflux process (Zhang et al 2022b) (Fig. 1). OsZIPl, as a metal-detoxified transporter, is located at the plasma membrane and endoplasmic reticulum, where it prevents excessive Cd and Zn accumulation in rice (Liu et al 2019a, b). Transgenic rice overexpressing *OsZIP1* exhibits better growth and accumulates fewer metals in plants under excessive Zn, Cu, and Cd stress (Liu et al 2019b). The *oszip1* mutant and the RNA interference (RNAi) line both exhibit increased metal ion accumulation in roots and increased sensitivity to metal stress, indicating that *OsZIP1* may function as a metal exporter of Zn, Cu, and Cd in rice (Liu et al 2019a, b). In contrast, *OsZIP3*-overexpressed transgenic rice plants display better growth performance under Cd stress and reduce the Cd concentrations in the roots and shoots (Li et al., 2023a). In *Arabidopsis thaliana*, the expression of *AtPDR8* increased significantly under Cd or Pb stress (Kim et al 2007). The *atpdr8* knockout plants and *atpdr8* RNAi plants show sensitivity phenotypes to Cd and Pb than WT under Cd stress, while the lines overexpressing *AtPDR8* are resistant to Cd with higher Cd extrusion (Li et al., 2023a; Kim et al 2007; Sheng et al 2019). These findings strongly confirm that AtPDR8 functions as an efflux pump of Cd^2+^ at the plasma membrane of *Arabidopsis* roots and leaves cells (Kim et al 2007). Additionally, OsABCG36 located at the plasma membrane contributes to the efflux of Cd in rice roots. The knockout of *OsABCG36* results in increased Cd accumulation and heightened sensitivity in the root sap cells (Li et al., 2023a).

#### Cd transport to shoots by loading into the Xylem

The Cd root-to-shoot transportation is fulfilled by Cd loading into the xylem vessel (Zhao et al 2022) (Fig. 1). The P1B-type ATPases, *AtHMA2* and *AtHMA4* expressed in the vascular tissues of roots, stems, and leaves are responsible for the root to shoot translocations of Cd and Zn in *Arabidopsis* (Verret et al 2004; Takahashi et al 2012). Overexpression of *AtHMA4* enhances Cd tolerance and increases Cd accumulation in stem than wild type. Conversely, the *athma4* mutant exhibits lower levels of Zn and Cd translocation from roots to shoot (Verret et al 2004). OsHMA2 is localized at the plasma membranes of the pericycle cells in the roots and the phloem region of the nodes. Knockout of *OsHMA2* significantly reduces Cd accumulation in shoots and grains in rice, suggesting that OsHMA2 is involved in Cd translocation from the roots to shoot (Tao & Lu 2022; Chang et al 2023). Zinc-regulated transporters and iron-regulated transporters like proteins 7 (OsZIP7) expressing in parenchyma cells of vascular bundles in roots and nodes are involved in Zn and Cd transport (Yang et al 2022b; Tan et al 2019). Knockout of *OsZIP7* enhances Zn and Cd retention effectively in the roots and basal nodes, preventing their transportations to upper nodes and brown rice (Zhao et al 2022; Tan et al 2019). As the other ZIP family member, OsZIP2 is also predicted to be located at the plasma membrane in root and shoot, involving the Cd root-to-shoot translocation (Li *et al.,* 2024).

CAL1 regulated Cd transportation via xylem located in the root and the leaf sheath xylem parenchyma cells, and *cal1* knockout mutant shows lower Cd concentration in leaves of rice under Cd treatment (Zhang et al 2022b; Li et al 2023). Metal Tolerance Protein 1 (OsMTP1) also plays an important role in Cd transportation and it is widely expressed in the root, stem, and leaf. The double-strand RNA interference (dsRNAi) plants of *OsMTP1* displays higher Cd concentration in root but lower Cd concentration in shoot than wildtype under Cd stress (Zhang et al 2022b; Sun et al 2023).

#### Cd Transport through the phloem to grains and leaves

In cereal crops, Cd transported from the xylem to the shoots is stored in the nodes. Nodes of cereal plants serve as a hub regulating the distribution of nutrients and Cd to leaves or reproductive organs through the phloem (Yoneyama et al 2010; Xia et al 2023) (Fig. 1). Several transporters have been identified to participate in these processes (Zhao et al 2022) (Fig. 1).

The low-affinity cation transporter (OsLCT1) has been identified as a phloem Cd transporter in plants mediating Cd efflux (Uraguchi et al 2011). *OsLCT1* is expressed in both the enlarged vascular bundles and the diffuse vascular bundles in rice (Zhang et al 2022b; Uraguchi et al 2014). Knockdown of *OsLCT1* reduces Cd concentration in the phloem sap and grains, indicating that *OsLCT1* possibly functions in Cd remobilization via the phloem and affects Cd translocation from shoots to grains (Uraguchi et al 2011). OsHMA2 and OsZIP7 are involved in Cd and Zn translocation in the root-to-shoot (Tan et al 2019; Xia et al 2023). Knockouts of *OsHMA2* or *OsZIP7* reduces Cd and Zn concentrations in rice grains, and Zn deficiency has a significant effect on plant growth and grain yield (Tan et al 2019; Takahashi et al 2012). *OsHMA3*, expressed in the tonoplast of root cells, is responsible for limiting Cd root-to-shoot transport in rice by Cd sequestration into root vacuole (Ueno et al 2010). *TdHMA3-B1* could also increase Cd accumulation in grain with a non-functional variant in durum wheat cultivar (Maccaferri et al 2019). The cation/Ca^2+^ exchanger 2 (OsCCX2) expressed in the xylem parenchyma cells of the stem node can load Cd into xylem vessels of diffuse vascular bundles and mediate Cd transportation into grains through the xylem (Hao et al 2018). Knockout of *OsCCX2* would decrease Cd accumulation in rice grains with a mild effect on grain yield (Hao et al 2018). Oligopeptide transporter 3 (OPT3) is expressed at the plasma membrane of the *Arabidopsis* phloem (Mendoza-Cózatl et al 2014; Zhai et al 2014). The transgenic *Arabidopsis* overexpressing *OPT3* reduces the Cd accumulation in grains and the *opt3* mutant *Arabidopsis* are hypersensitive to Cd (Mendoza-Cózatl et al 2014).

### Repair the cellular damage under Cd stress

**Fig. 2:**
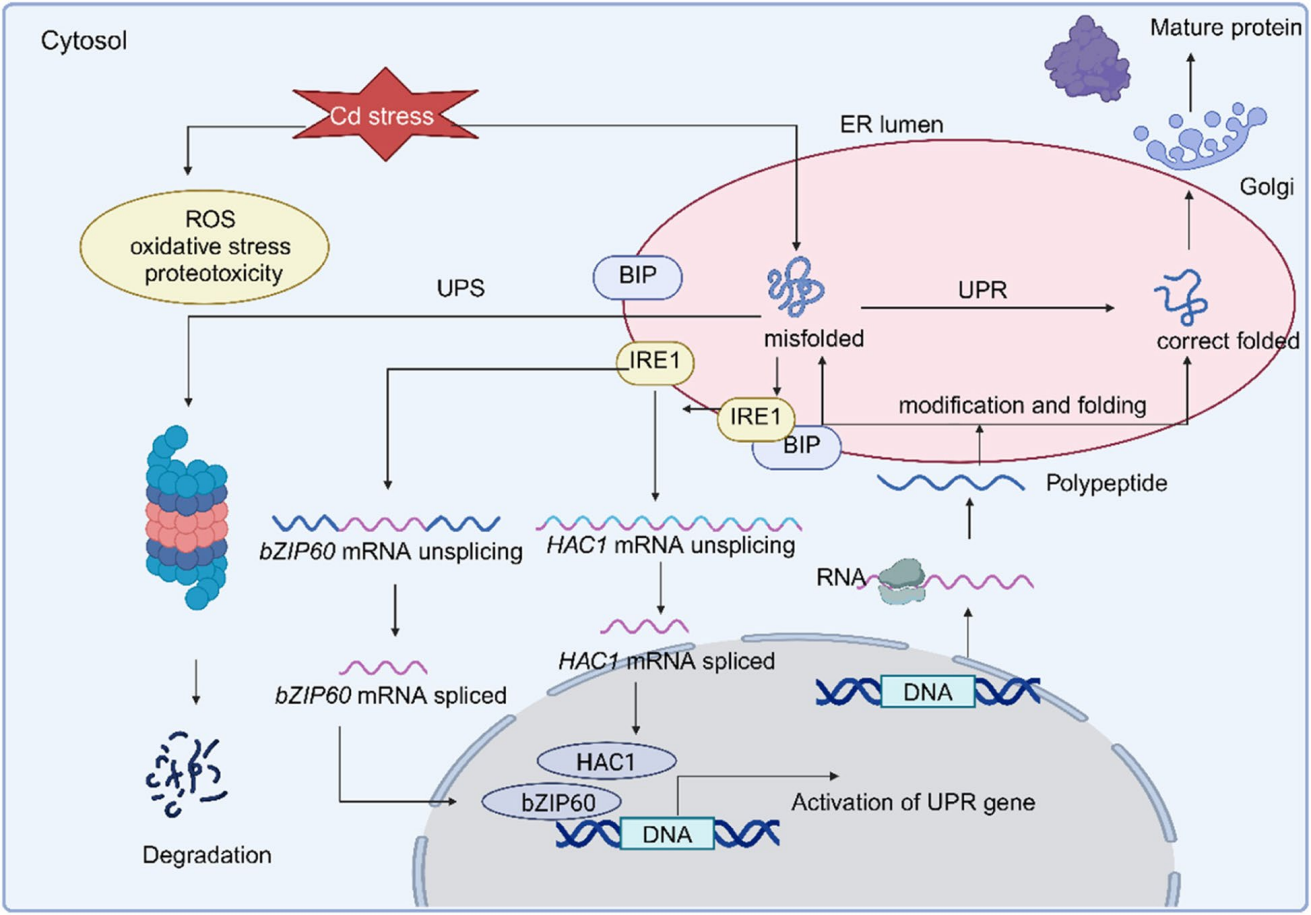
Cd stress resulted in ROS production and disturbed the structural of protein, which induced the aggregation of misfolded proteins and ER stress. To alleviate the ER stress, the Unfolded Protein Response (UPR) and ER-associated degradation (ERAD) systems are initiated to promote the correct folding of misfolded proteins and remove the misfolded proteins. ER stress induced the disassociation of IRE1 from BIP, and IRE1 caused the splicing of *bZIP60* and *HAC1* mRNA, which are transcription factors to active UPR response genes

Cadmium toxicity brings cellular damage to plants. One of the Cd toxicity effects in plants is generation of reactive oxygen species (ROS), such as H_2_O_2_, O^2−^, and OH^−^ (Unsal et al., 2020). Furthermore, it induces lipid peroxidation and the excess of malondialdehyde (MDA) (Anjum et al 2015). Free radicals decrease the reducing equivalents, such as nicotinamide adenine dinucleotide phosphate (NADPH) participating in redox reactions for photo-assimilation. The deleterious effects of Cd on photosynthetic pigments and photosystems significantly inhibit plant growth under Cd stress (Demirevska-Kepova et al 2006). In addition, Cd impairs the functions of proteins by substituting metal cofactors like Zn and Ca ions and changing the structure of proteins. Cd prevents the refolding of chemically denatured proteins in vitro causing the accumulation of protein aggregates in vivo (Ozturk et al 2021). More polypeptides may not be correctly folded as a result of the protein aggregates, which causes more proteins to aggregate, disturbing the overall cellular homeostasis and ultimately leading to cell death (Ozturk et al 2021; Roychoudhury 2021) (Fig. 2). For instance, the activities of photo assimilation related enzymes, such as ribulose-1,5 bisphosphate carboxylase/oxygenase (RUBISCO), and nitrate reductase are inhibited under Cd stress, which would ultimately reduce the biomass of plants (El Rasafi et al 2022).

#### Antioxidant defense system

The antioxidant defense system helps to maintain the redox balance of the plant cells by transforming ROS into less harmful chemicals (Patra et al 2011) (Fig. 2). The ROS are scavenged by the enhanced antioxidant activities of superoxide dismutase (SOD), peroxidase (POD), catalase (CAT), ascorbate peroxidase (APX), and glutathione peroxidase (GPX) in response to Cd stress in *Zea mays L.* (Anjum et al 2015). The *ZmWRKY4* gene in maize may control the activity of SOD and APX under Cd stress (Hong et al 2017). Transgenic tobacco overexpressing *ZmVTE4*, which encodes c-tocopherol-methyl-transferase, increased the amount of c-tocopherol and a-tocopherol, enhancing the tobacco resistance to Cd stress (Zhu et al 2019). Transgenic *Arabidopsis thaliana* overexpressing glutaredoxin derived from chickpea leaves enhanced the Cd tolerance, increasing the plants growth and enhancing the activities of antioxidants enzymes such as GPX, APX, SOD, and CAT (El Rasafi et al 2022).

#### Cd chelation and sequestration of vacuolar

Another biological process impacting Cd accumulation is the chelation between Cd and metal-binding peptides, which is involved in the Cd subcellular distribution and translocation with the help of specific transporters (Peng & Gong 2014). Glutathione (GSH), phytochelatins (PCs), and metallothioneins (MTs) are common ligands for binding Cd in plants(Yang et al 2022b) (Fig. 1). For example, yeast cadmium factor 1 (*YCF1*) encoding a vacuolar glutathione S-conjugate pump can transport Cd-GS_2_ complex into the vacuoles, restricting the Cd mobility and alleviating the Cd toxicity in plants. PCs synthesized by PC synthetase (PCS) from GSH are involved in Cd remobilization and storage, as well as regulating Cd xylem-to-phloem translocation (Yang et al 2022b). Moreover, a loss-of-function mutant of YCF1 named *ycf1* can enhance the Cd resistance via overexpression of *OsPCS5* and *OsPCS15* (Park et al., 2019). *Arabidopsis cad1* mutant lacking PC synthase activity, is hypersensitive to Cd (Cobbett et al 1998). Rice expressing *TaPCS1* of the wheat had higher Cd sensitivity and higher Cd deposition in shoots compared to roots (Zhang et al 2022b; Wang et al 2012). Furthermore, transcription factor *OsHsfA4a* can increase Cd resistance in rice by inducing the expression of the metallothionein (MT) gene and the chelation of MT and Cd (Zhang et al 2022b; Li et al., 2023a).

Vacuolar sequestration is another mechanism limiting Cd translocation in plants. Several alleles of heavy metal ATPase 3 (*OsHMA3*) have been linked to high Cd accumulation in rice grains by QTL mapping (Pan et al 2020). *OsHMA3* encodes a tonoplast-localized P1B-type heavy metal ATPase, facilitating the Cd sequestration in vacuoles and decreasing Cd mobility (Miyadate et al., 2011). Overexpression of *OsHMA3* reduces Cd accumulation in the shoot and increases Cd concentration in the roots selectively, indicating overexpression of *OsHMA3* enhances vacuolar sequestration of Cd in the roots (Sasaki et al 2014). Apart from *OsHMA3*, the Cd vacuolar sequestration in rice roots is also carried out by a C-type ABC transporter (OsABCC9), which is situated at the tonoplast in the parenchyma cells inside the stele (Ai et al., 2022). The expression of *OsABCC9* increased in rice roots under Cd stress and knockout of *OsABCC9* leads to increased Cd accumulation in rice xylem sap and grains (Yang et al 2021).

#### Misfold protein

Heavy metal stress would lead to the accumulation of the misfolding proteins. Endoplasmic reticulum (ER) stress occurs as misfolded proteins accumulate in the ER (Ozturk et al 2021; Manghwar & Li 2022; Le et al 2016). The unfolded protein response (UPR) is initiated through transcriptional and translational events to mitigate ER stress (Read & Schröder 2021; Liu & Kaufman 2003) (Fig. 2). UPR aims to restore the normal functioning of the cell by inhibiting the production of secreted and membrane proteins, eliminating the misfolded proteins via ER-associated degradation (ERAD) systems, and triggering the signaling pathways to generate more molecular chaperones involved in protein folding (Roychoudhury 2021; Hetz et al 2020; Hoseki et al 2010; Nishikawa et al 2005). For example, the HSP70 chaperone, also known as binding immunoglobulin protein (BIP), tags the misfolded or unfolded proteins and induces the UPR (Wang et al 2017). Accumulation of unfolded proteins leads to ER stress, causing BIP disassociation from the stress sensors inositol-requiring enzyme 1(IRE1) (Le et al 2016; Deng et al 2011). Stress sensors embedded in the ER membrane like IRE1 further regulate the activation of the downstream signaling pathway (Le et al 2016; Zhang et al 2019) (Fig. 2). IRE1, undergoing oligomerization, followed by autophosphorylation of the cytosolic kinase domains, would cause the unconventional splicing of basic leucine zipper 60 (bZIP60) mRNA (Li et al 2012; De Benedictis et al 2023). Spliced and mature *bZIP60* encodes an active transcription factor, triggering the expression of UPR genes, including heat shock proteins (Roychoudhury 2021; De Benedictis et al 2023; Manghwar & Li 2022) (Fig. 2). In addition, IRE1 cut off the intron from the mRNA of the *HAC1* gene, which is a transcription factor responsible for the transcription of UPR genes (Le et al 2016; Gardarin et al 2010; Ozturk et al 2021; Zhao et al 2021) (Fig. 2). An extensive family of molecular chaperones known as heat shock proteins (HSPs) are induced in stress response, help misfolded proteins fold correctly, and stop them from aggregating or selectively degrading (Roychoudhury 2021; Hasan et al 2017). For example, all the nascent polypeptides before properly modified and folded, are stabilized by chaperones (HSP40 and HSP70-like proteins) such as ERdj3 and binding protein (BIP) in ER (Hasan et al 2017).

The majority of aberrant peptides and short-lived cellular regulators are eliminated by the ubiquitin proteasome system (UPS), which operates in the cytoplasm and nucleus and is in charge of regulating the levels of regulatory proteins (Hasan et al 2017). The E3 or ubiquitin ligase enzymes are crucial regulators for the elimination of aberrant proteins under metal stress (Lyzenga & Stone 2012) (Fig. 2). For example, the RING E3 ligase 1 (*OsHIR1*) in *Oryza sativa* is significantly upregulated under arsenic (As) and Cd treatments, which interacts with tonoplast intrinsic protein 4;1 (OsTIP4;1) in the plasma membrane and degrades OsTIP4;1 via the ubiquitin 26S proteasome system (Lim et al 2014; Liu et al 2022). Overexpression of *OsHIR1* in *Arabidopsis* increases As and Cd tolerance and decreases As and Cd accumulation in the shoots and roots compared with the control (Lim et al 2014). These cellular responses could increase the Cd tolerance via the removal of misfolded proteins.

## Ways to enhance Cd tolerance and decrease the Cd accumulation in edible parts of crops

### Breeding of low Cd accumulation crops

**Fig. 3:**
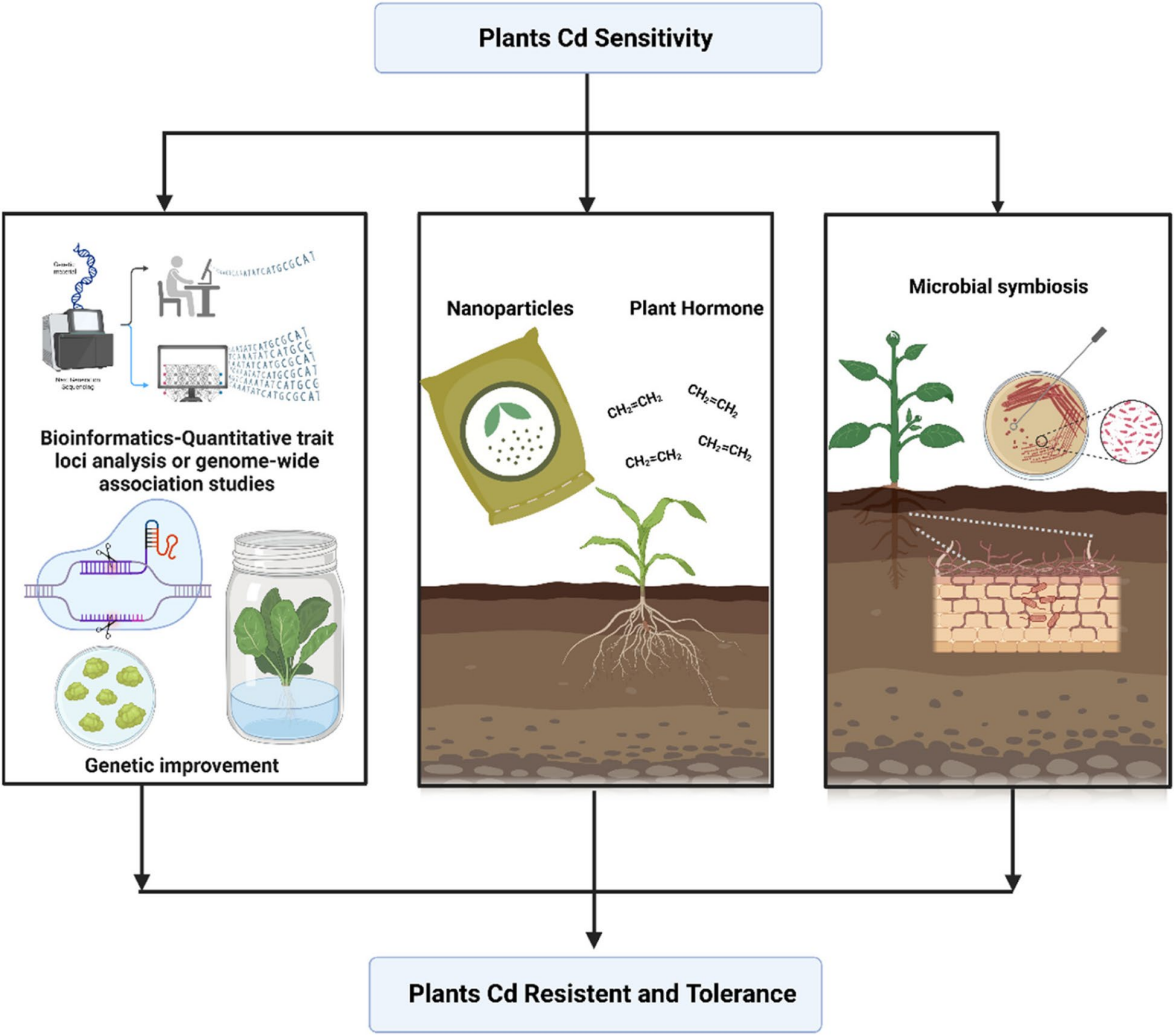
Genetic–chemical materials–root microbe strategy for improving Cd stress resistance in crops. Genetic and molecular studies have improved our understanding of Cd stress responses in plants. High throughput sequencing and bioinformatics analysis identified natural allelic variants and candidate genes representing low Cd accumulation. CRISPR–Cas-based gene editing can be applied in breeding plants with Low Cd accumulation and Cd tolerance. Chemical materials, like nano-particles, are potential methods for manipulation to protect crops from Cd stresses at multiple levels. The inoculation with beneficial microbes in plants roots can increase Cd tolerance and decrease Cd uptake and translocation

The molecular mechanism for Cd accumulation in crops has not been clearly elucidated. Natural allelic variants representing low Cd accumulation have been distinguished in crop species, via quantitative trait loci (QTL) mapping, bulked segregant analysis (BSA), and genome-wide association studies (GWAS), which can be applied in breeding Cd-PSC crops (Pan et al 2020; Zhao et al 2018b) (Fig. 3). For example, the *OsHMA3* involved in Cd root-to-shoot transport and Cd accumulation in grains is identified in a QTL using quantitative trait loci mapping (Sui et al 2019; Sun et al 2019). A major QTL of *Gr_Cd_Conc*−4B is screened by QTL mapping, which consists of candidate gene encoding Cation/Ca exchanger 2 (TpCCX2), and explaining 26.64% to 31.89% of the grain Cd variation in polish wheat. Overexpression of *TpCCX2-4B* reduced Cd concentrations of grains in rice (Cheng et al 2024). For example, CAL1, identified by QTL mapping, regulates Cd accumulation positively by encoding a defensin-like protein in rice leaves (Luo et al 2018). The BSA facilitated the QTL mapping by the separated populations with similar genetic background and extreme phenotypes. With the help of BSA, nine candidate QTLs from five chromosomes were identified in the delta SNP index between the two bulked pools of 50 individuals with high Cd accumulation and 50 individuals with low Cd accumulation, respectively (Wang et al 2023a). The Cd-related loci/genes QTL mapped or cloned in crops have been summarized in Table 1.

**Table 1:** Summaries of Cd-related loci/genes QTL mapped or cloned in crops for molecular breeding of low-Cd crops

QTL	Gene	Functions	Species	References
*qCd7*1 *QGr_Cd_Conc-4B* 1 *QTL3* *qLCd2* *QCd.uia2-7B* *qCd1-3* *qCd3–2* *qCd1*	*OsHMA3* *TpCCX2-4B* *OsCd1* *OsCAL1* *TaHMA2* *OsABCB24* *OsNRAMP2* *ZmHMA3*	Cd sequestration from the cytoplasm into the vacuoles of root cells Endoplasmic reticulum membrane/plasma membrane-localized Cd efflux transporter Root Cd uptake and grain accumulation A defensin-like protein by chelating Cd in the cytosol and facilitating Cd secretion to extracellular spaces grain Cd content Cd accumulation Cd accumulation A tonoplast-localized heavy metal P-type ATPase transporter grain Cd accumulation	*Oryza sativa* *Triticum polonicum* *Oryza sativa* *Oryza sativa* *Triticum aestivum* *Oryza sativa* *Oryza sativa* *Zea mays L*	(Sui et al 2019) (Cheng et al 2024) (Yan et al 2019) (Luo et al 2018) (Qiao et al 2021) (Pan et al 2020) (Zhao et al 2018a) (Tang et al 2021)

Most of the Cd-related QTLs were identified using bi-parental populations with limited allele diversity and less recombination, which has hindered the progress of QTL mapping (Pan et al 2020). GWAS can overcome these limitations and perform as a powerful tool to identify genome regions associating with Cd accumulation traits. Through a GWAS of 276 diverse accessions, 60 QTLs were detected for accumulation of As, Cd, and Pb in rice (Liu et al 2019a). 14 QTLs associated with Cd accumulation are unraveled in rice by GWAS, and *OsNRAMP2* is predicted as the candidate gene of *qCd3–2* (Zhao et al 2018a). By using GWAS analysis on 338 different rice accessions, a novel QTL qCd1 − 3 with the candidate gene *OsABCB24* has been identified to be associated with low Cd accumulation (Pan et al 2020). In a GWAS analysis using 513 maize inbred lines, the candidate gene *ZmHMA3* is found to be a major locus controlling Cd content in maize grain (Cao et al 2019). These studies suggest that the identification of major alleles associated with Cd accumulation and the discovery of Cd-resistant germplasm resources of crops could provide new genes and raw materials for the breeding of low-Cd accumulating crop cultivars (Hu et al 2022). These findings are helpful for further exploiting novel functional genes and underlying molecular mechanisms related to Cd accumulation, which also provides a useful target for breeding crop cultivars with low-Cd accumulation through marker-assisted breeding.

Molecular breeding practices, such as genome editing, transgenic technology, and marker-assisted selection (MAS) accelerate the progress of breeding low Cd accumulative cultivars for crop improvement (Ni et al 2023; Zhang et al 2022a) (Fig. 3). CRISPR-Cas9 is applied to decrease Cd accumulations by knocking out Cd uptake and transporter genes (Ni et al 2023). The knocked out of *OsNramp5* in rice decreased Cd concentration in grain (< 0.05 mg/kg) (Tang et al 2017). Knocking out the node-expressed transporter gene *OsCCX2* in rice would significantly reduce Cd accumulation in grains, and root to shoot Cd transportation ratio (Hao et al 2018). Two *Ospmei12* mutant lines obtained from CRISPR/Cas9 have decreased the pectin methylation and increased pectin content in *Nipponbare* under Cd stress, indicating that *OsPMEI12* is a potential locus for controlling Cd accumulation in *Nipponbare* (Li et al 2022). Although the gene editing technology has accelerated the understanding of Cd uptake and accumulation mechanism, the development of Cd-PSC is still insufficient. The selection of mutated genes needs to be examined thoroughly, since most genes have involved in multiple pathways for plant growth and metabolism (Yang et al 2022b). In the further investigation of Cd-PSC breeding, the potential harmful effects of gene knockout should also be considered, which are detrimental to crop growth and threaten human health.

With genetic engineering tools, overexpressing *SbMYB15* of *Salicornia brachiata* in transgenic tobacco can reduce the Cd absorption (Sapara et al 2019). Overexpression of *miR166* reduced both Cd translocation from roots to shoots and Cd accumulation in the grains and improved Cd tolerance (Ding et al 2018), which could be partly ascribed to the down-regulation of *HOMEODOMAIN CONTAINING PROTEIN4 (OsHB4)* in rice (Li et al., 2023b; Ding et al 2018). The wheat overexpresses *OsHMA3* sequestrating Cd in roots, which results in 110–125% higher Cd content in roots while reducing Cd content in wheat grains by up to 40 times (Zhang et al 2020b; Zulfiqar et al 2022). This method provides a practical solution to reduce Cd content in crops.

The reliable molecular markers such as Single nucleotide polymorphisms (SNPs) could be applied in selecting individuals with the help of MAS. An SNP in the promoter of *OsHMA3* is responsible for the different Cd accumulations between *indica* and *japonica* rice varieties (Liu et al 2020). DNA marker of *OsNRAMP5* has facilitated marker-assisted selection of cultivars carrying *osnramp5* to screen low Cd accumulation cultivars (Ishikawa et al 2012; Ni et al 2023). The low-Cd accumulation rice cultivar *Pokkali* results from the duplication of a gene encoding a manganese/Cd transporter. Introgression of *OsNramp5* allele from low-Cd accumulation rice cultivar into *Koshihikari* significantly reduces Cd accumulation in the grain (Yu et al 2022). In conclusion, the MAS could overcome the limitations of conventional breeding methods and accelerate the selection of Cd-PSCs with the help of efficient DNA marker identification (Ni et al 2023).

### Interactions of chemical materials and Cd in plant systems

Chemical materials have been applied to maintain the Cd accumulation for a long term. For example, nutrient elements like selenium (Se) and boron (B) regulated cell wall functions, which could immobilize Cd in cell wall polysaccharide components in rice and *Capsicum annuum* (Wang et al 2023b; Huang et al 2022) (Fig. 3). Besides, NO addition could promote hemicellulose biosynthesis and de methyl esterification of pectin, both of which effectively enhance Cd immobilization in the root cell wall and reduce Cd translocation (Yang et al 2022a). Recently, with the advancements of nano-materials, studies have investigated the potential of different nano-materials in lowering the Cd accumulation in crops. For instance, exposure to polystyrene nano-plastics (PS NPs) in maize could reduce Cd-induced toxicity owing to the antagonistic effect and surface adsorption (Wang et al 2022). Astaxanthin and its gold nanoparticles (AstNPs) reduce Cd toxicity by blocking its uptake and translocation through the regulation of transporter genes and activation of the antioxidant system in rice (Hu et al 2022; Soni et al 2024). NPs-Ca influences the transcription of *PME*, *PMEI*, and *PG* related genes, reducing the activity of pectin-degrading enzymes, and promoting the activity of PME, which significantly increases 59.8% of Cd accumulated in cell wall (Zhu et al 2023). Nanoparticles offer enormous potential for soil remediation, while Cd removal using nanoparticles has not been used extensively due to safety concerns (Hu et al 2022).

### Microbial strategy for improving Cd stress resistance in crops

Beneficial soil microbes are unraveled in enhancing plant resistance to Cd stress and productivity (Zhang et al 2022a) (Fig. 3). An endophytic *Fusarium* of Fo10 is isolated, which is sensitive to Cd efflux protein OsCAL1 in rice and tolerant to high doses of Cd (Gu et al 2024). *Arabidopsis thaliana* gene was inserted into *Mesorhizobium huakuii* subsp. *Rengei* strain B3 for PCS, which would increase binding Cd^2+^ by 9- 19 folds (Sriprang et al 2003). *Rhizophagus intraradices* belonging to AMF could enhance shoot biomass and reduce the Cd root-to-shoot transportation in rice by cell wall remodeling (Gao et al 2021). Taken together, these findings provide insights into the interaction mechanisms between microbes and host plants to reduce Cd accumulation in crops (Gu et al 2024).

## Conclusions and future perspectives

Cd exposure and accumulation in crops induce serious risks to human health. Breeding new low Cd accumulating cultivars to decrease the Cd contamination risk in plants is an economical and environmentally friendly approach. We summarized extensive mechanisms of Cd uptake, translocation, sequestration, and detoxification processes. Different Cd tolerance genes have been found in plants over the past decades, and their functional analysis has offered vital insights regarding the breeding of Cd-PSC by molecular and genetic approaches. It is urgent to identify key target genes with improved high-throughput sequencing and gene editing techniques, which can offer insightful information about the fundamental processes of Cd tolerance and homeostasis for breeding Cd-PSC. Long-term field experiments are necessary for estimating the risks and benefits of various amendments for food safety and human health, whereas in-depth research is required to understand how different amendments reduce the toxicity of Cd in plants (Zulfiqar et al 2022). In conclusion, a combination of molecular genetics methods, chemical materials, and microbial approaches will contribute greatly to our knowledge about responding mechanisms to increase Cd tolerance and reduce Cd accumulation in crops, ultimately ensuring sustainable agricultural practices and food safety (Zhang et al 2022a).

## Data Availability

No datasets were generated or analyzed during the current study.
